# Supplementary material for: Diet-Wide Association Study for the Incidence of Type 2 Diabetes in Three Population-Based Cohorts
Source: Nutrients. 2024 Nov 5;16(22):3798. doi: 10.3390/nu16223798 (PMC11597135; doi:10.3390/nu16223798)

Supplementary Table S1. Description of 47 foods and 62 nutrients

No.	Foods and Nutrients	*Abbreviation*	No. of food items*	Food items
FOODS (Serving/day)				
Food1	Cooked white rice	*WhiteRice*	1	Cooked white rice
Food2	Cooked rice with beans	*RiceBeans*	2	Cooked rice with beans
Food3	Cooked rice with multi-grains	*MultiGrainRice*	2	Cooked rice with multi-grains
Food4	Noodles	*Noodles*	4	Ramyon, Kalguksu/Jangguk-noodles/Udon, Chajangmyon/ Champpong, Naengmyeon/Buckwheat noodles
Food5	Dumpling and Tteokguk	*DumpTteok*	2	Dumpling/Dumpling soup, plain stick shaped-rice cake/Tteokguk (plain stick shaped-rice cake soup)
Food6	Rice cakes	*RiceCakes*	1	Other rice cakes (Steamed rice cake, Baekseolgi, Injeolmi, etc.)
Food7	Cornflakes	*Cornflake*	1	Cornflakes
Food8	Breads/spreads	*BreadSpread*	2	Loaf bread/sandwich/toast, jam/honey/margarine
Food9	Bread products	*BreadProducts*	3	Bread with small red bean, other breads (streusel bread/nut cake/castella/cream bread), cake /chocopie
Food10	Cheese and pizza/hamburger	*ChzPizzaBurger*	2	Pizza/hamburger, cheese
Food11	Grain powder	*GrainPowder*	1	Powdered meals/parched cereal powder
Food12	Snack/confections	*SnackConfect*	1	Cookie/cracker/snack, candy/chocolate
Food13	Nuts and roasted beans	*NutsRoastBeans*	2	Peanut/almond/pine nut, soybeans/soybeans cooked in soy sauce (include green beans, exclude rice with beans)
Food14	Soybean paste soup	*SoybeanSoup*	1	Soybean paste soup/fast-fermented bean paste/soybean paste/ Ssamjang
Food15	Tofu/bean sprouts	*TofuSprouts*	2	Tofu (soft tofu, tofu stew, and tofu in tofu stew), bean sprouts/mung bean sprouts
Food16	Eggs	*Eggs*	1	Egg/quail egg
Food17	Starch	*Starch*	2	Muk (starch jelly), Japchae (starch vermicelli)
Food18	Potatoes	*Potatoes*	2	Potatoes (steamed, French fries, soup, stew, jeon, etc.), sweet potatoes (steamed sweet potatoes, Mattang, etc.)
Food19	Baechukimchi	*Baechukimchi*	1	Baechukimchi/Baegkimchi/Baechukimchi in Kimchi stew
Food20	Other kimchi	*OtherKimchi*	3	Kkakdugi/Radish Kimchi, Nabakkimchi/Dongchimi, Other Kimchi (Pakimchi/Kodulbbagi/Gatkimchi)
Food21	Salt-fermented food	*SaltFermented*	2	Other pickled vegetables (garlic pickles, garlic stems, radish pickles), salt-fermented fish (salted squid, salted intestine, salted pollack roe, salted shrimp, salted anchovies, salted clams, etc.)
Food22	Cabbage/radish Soup	*CabbageRadishSoup*	2	Radish (soup, stew)/pickled radish, cabbage/cabbage soup
Food23	Vegetable dish	*VegDish*	7	Spinach (spinach namul, soup, etc.), vegetable wraps/vegetable salad (cabbage, lettuce, kale, chicory, bok choy, broccoli, etc.), other green vegetables (shepherd's purse, beetroot, curled mallow, mugwort, outer leaves, etc.), Doraji/Deoduck (kind of white root), bracken/sweet potato stems/taro stem, red pepper leaves/Chamnamul/Chwinamul, crown daisies/leek/water dropwort, carrot/carrot juice
Food24	Lettuce/perilla leaf	*LettucePerilla*	2	Lettuce (Ssam (rice and condiments wrapped in leaves of lettuce)/salad, etc.), perilla leaf
Food25	Mushrooms	*Mushrooms*	2	Oyster mushroom (Pleurotus ostreatus), Other mushrooms (Wood ear mushroom (Auricularia heimuer), Button mushroom (agaricus bisporus), Winter mushroom (flammulina velutipes) etc.))
Food26	Other vegetables	*OtherVegetables*	4	Cucumber, Onion, Green pepper, Pumpkin, immature
Food27	Sweet pumpkin	*SweetPumpkin*	1	Pumpkin/Sweet pumpkin/Pumpkin juice
Food28	Poultry	*Poultry*	1	Fried chicken/Whole Chicken Soup/Samgyetang/Chicken Stew
Food29	By-products (organ meat, Seonji, Sundae)	*OrganMeatByproduct*	1	By-products (Organ meat, Seonji, Sundae)
Food30	Cuttlefish	*Cuttlefish*	1	Cuttlefish/Dried cuttlefish/Small octopus
Food31	Pork	*Pork*	3	Pork belly, Pork-Pan roasted/Fried/Pork bulgogi/Meatball, Pork-steamed (Boiled pork, pork braised in soy sauce, pigs' feet)
Food32	Processed meat/seafood	*ProcessedMeatSeafood*	3	Processed meat (Ham, sausage), Tuna, canned, Fish paste/Crab, flavored.
Food33	Beef	*Beef*	3	Steak/beef roast (Grilled ribs, Sirloin, Tenderloin, Beef bulgogi), Tang (Seolleongtang/Gomtang/Galbitang/ Doganitang), Soup (Beef soup, Yukgaejang, etc.)
Food34	Sliced raw fish and eel (special fish)	*RawFishEel*	2	Sliced Raw Fish, Eel
Food35	Dog meat	*DogMeat*	1	Dog meat
Food36	Fish	*Fish*	4	Blue-colored back fish (Mackerel/Pacific saury/Spanish mackerel), Hair tail, Yellow croaker/Snapper/Halibut, Alaska pollack/Frozen Alaska pollack/Dried Alaska pollock
Food37	Anchovy	*Anchovy*	1	Dried anchovy/Stir-fried dried anchovies
Food38	Seaweed	*Seaweed*	2	Laver-dried, Kelp/Sea mustard
Food39	Shellfish seafood	*ShellfishSeafood*	4	Clam (Small ark shell/Little neck clam/Clam meat)/Whelk (including Soup, Stew, Roast, Kalguksu, Salad, etc.), Oysters (including Salted oysters), Crab/Crab preserved in soy sauce, Shrimp
Food40	Milk/yogurt	*MilkYogurt*	2	Milk, Yogurt/Yoplait
Food41	Carbonated drink/ice cream	*CarbDrinkIcecream*	2	Ice cream, Carbonated drinks (Coke, Sprite)
Food42	Soy milk	*SoyMilk*	1	Soy milk
Food43	Coffee	*Coffee*	3	Coffee, Coffee Sugar, Coffee Cream
Food44	Traditional beverages	*TradBeverages*	1	Other beverages (Citron tea, Plum tea, Aloe, Persimmon punch, Ginseng tea, Sikhye, Jujube tea, Black herbal tea, etc.)
Food45	Green tea	*GreenTea*	1	Green tea
Food46	Native fruit	*NatFruit*†	10	Korean melon/Melon, Watermelon, Peach/Plum, Persimmon, hard/Persimmon, dried, Tangerine, Korean pear/Pear juice,

				Apple/Apple juice, Grapes/Grapes juice, Tomato/Tomato juice/Cherry tomato
Food47	Non-native fruit	*NonNatFruit*‡	2	Banana, Orange/Orange juice
NUTRIENTS				
Nutri1	Carbohydrates, g	*CHO*		
Nutri2	Fat, g	*FAT*		
Nutri3	Vegetable fat, g	*Fat_veg*		
Nutri4	Animal fat, g	*Fat_ani*		
Nutri5	Protein, g	*PRO*		
Nutri6	Vegetable protein, g	*Pro_veg*		
Nutri7	Animal protein, g	*Pro_ani*		
Nutri8	Dietary fiber, g	*Fiber*		
Nutri9	Vitamin A, ug RE	*VitA*		
Nutri10	Vitamin D, ug	*VitD*		
Nutri11	Vitamin K, ug	*VitK*		
Nutri12	Thiamine, mg	*VitB1*		
Nutri13	Riboflavin, mg	*VitB2*		
Nutri14	Niacin, mg	*Niacin*		
Nutri15	Vitamin B6, mg	*VitB6*		
Nutri16	Folic acid, ug	*Folate*		
Nutri17	Vitamin B12, ug	*VitB12*		
Nutri18	Pantothenic acid, mg	*VitB5*		
Nutri19	Biotin, ug	*VitB7*		
Nutri20	Calcium, mg	*Ca*		
Nutri21	Vegetable calcium, mg	*Ca_veg*		
Nutri22	Animal calcium, mg	*Ca_ani*		
Nutri23	Phosphorus, mg	*P*		
Nutri24	Sodium, mg	*Na*		
Nutri25	Chlorine, mg	*Cl*		
Nutri26	Potassium, mg	*K*		
Nutri27	Magnesium, mg	*Mg*		
Nutri28	Iron, mg	*Fe*		
Nutri29	Vegetable iron, mg	*Fe_veg*		
Nutri30	Animal iron, mg	*Fe_ani*		
Nutri31	Zinc, mg	*Zn*		
Nutri32	Copper, mg	*Cu*		
Nutri33	Fluorine, ug	*F*		
Nutri34	Manganese, mg	*Mn*		
Nutri35	Iodine, ug	*Iodine*		
Nutri36	Selenium, ug	*Se*		
Nutri37	Cholesterol, mg	*Chol*		
Nutri38	Phytate, mg	*Phytate*		
Nutri39	Cereal fiber, g	*cFiber*		
Nutri40	Vegetable fiber, g	*vegeFiber*		
Nutri41	Fruit fiber, g	*fruitFiber*		
Nutri42	Glycemic load	*GL*		
Nutri43	Monounsaturated fatty acid, g	*MUFA*		
Nutri44	Polyunsaturated fatty acid, g	*PUFA*		
Nutri45	n-3 Polyunsaturated fatty, g	*n3_PUFA*		
Nutri46	n-6 Polyunsaturated fatty, g	*n6_PUFA*		
Nutri47	Saturated fatty acid, g	*SFA*		
Nutri48	Soy protein, g	*Pro_soy*		
Nutri49	Glycemic index,	*GI*		
Nutri50	Isoleucine, leucine, valine, mg	*AA_BCAAs*		
Nutri51	Retinol, ㎍	*Retinol*		
Nutri52	Vitamin C, mg	*VitC*		
Nutri53	Vitamin E, mg	*VitE*		
Nutri54	Carotenoids, ㎍	*Carotenoids*		
Nutri55	Flavonols, mg	*Flavonols*		
Nutri56	Flavones, mg	*Flavones*		
Nutri57	Flavones, mg	*Flavanones*		
Nutri58	Flavan-3-ols, mg	*Flavan3ols*		
Nutri59	Anthocyanins, mg	*Anthocyanins*		
Nutri60	Isoflavones, mg	*Isoflavones*		
Nutri61	Proanthocyanidins, mg	*PA*		
Nutri62	Total flavonoids, mg	*FLA*		

*At baseline in the combined Cardiovascular Disease Association Study (CAVAS) cohort, we consolidated 106 food items into 104 by merging related rice foods. Five rice-related items were condensed into three (Cooked white rice, Cooked rice with beans, Cooked rice with multi-grains). We then applied Ward's hierarchical clustering method with the Silhouette method to determine optimal clusters, considering predefined cluster numbers (20, 30, 40). After examining cluster interpretability and consistency, we ultimately identified 47 modified food groups.

† Native fruit referred to fruits primarily grown and consumed in Korea.

‡Non-native fruit referred to fruits not traditionally cultivated in Korea but imported for consumption.

Supplementary Table S2. Age- and sex-adjusted mean (standard error) dietary factors across the three cohorts.

47 Foods and 62 Nutrients*	*MRCohort*		*ARIRANG*		*Kanghwa*		*P* difference
	Mean	SE	Mean	SE	Mean	SE	
Foods (serving /day)							
Cooked white rice	1.20	0.013	1.10	0.017	0.77	0.021	<.0001
Cooked rice with beans	0.29	0.008	0.27	0.011	0.53	0.013	<.0001
Cooked rice with multi-grains	1.28	0.013	1.38	0.016	1.40	0.019	<.0001
Noodles	0.17	0.002	0.20	0.002	0.16	0.003	<.0001
Dumpling and Tteokguk	0.02	0.001	0.06	0.001	0.02	0.001	<.0001
Rice Cakes	0.03	0.001	0.04	0.001	0.04	0.001	<.0001
Cornflake	0.004	0.0004	0.004	0.0005	0.005	0.0006	0.5104
Breads/Spreads	0.06	0.002	0.05	0.002	0.06	0.003	0.0808
Bread Products	0.07	0.001	0.08	0.002	0.09	0.002	<.0001
Cheese and Pizza/hamburger	0.02	0.001	0.03	0.002	0.03	0.002	<.0001
Grain Powder	0.05	0.001	0.03	0.002	0.04	0.002	<.0001
Snack/Confections	0.10	0.002	0.09	0.003	0.10	0.004	0.0032
Nuts and Roasted beans	0.14	0.003	0.16	0.004	0.15	0.005	<.0001
Soybean paste soup	0.51	0.005	0.55	0.007	0.31	0.008	<.0001
Tofu/Bean sprouts	0.34	0.004	0.45	0.005	0.35	0.006	<.0001
Eggs	0.18	0.003	0.19	0.004	0.19	0.004	0.1233
Starch	0.03	0.001	0.04	0.001	0.05	0.001	<.0001
Potatoes	0.30	0.004	0.37	0.005	0.33	0.007	<.0001
Baechukimchi	1.91	0.012	1.93	0.016	2.11	0.019	<.0001
Other Kimchi	0.80	0.013	1.04	0.016	0.89	0.019	<.0001
Salt-fermented food	0.14	0.003	0.18	0.004	0.19	0.005	<.0001
Cabbage/Radish Soup	0.23	0.004	0.35	0.005	0.22	0.006	<.0001
Vegetable dish	0.40	0.006	0.56	0.008	0.53	0.009	<.0001
Lettuce/Perilla leaf	0.43	0.005	0.36	0.006	0.42	0.008	<.0001
Mushrooms	0.11	0.002	0.15	0.003	0.14	0.004	<.0001
Other vegetables	1.15	0.012	1.05	0.015	1.49	0.018	<.0001
Sweet pumpkin	0.03	0.001	0.04	0.002	0.04	0.002	<.0001
Poultry	0.02	0.001	0.03	0.001	0.03	0.001	<.0001
By-products (Organ meat, Seonji, Sundae)	0.02	0.0004	0.02	0.001	0.02	0.001	0.0004
Cuttlefish	0.03	0.001	0.03	0.001	0.03	0.001	<.0001
Pork	0.11	0.001	0.12	0.002	0.13	0.002	<.0001
Processed meat/seafood	0.05	0.001	0.06	0.002	0.06	0.002	0.1017
Beef	0.10	0.002	0.07	0.002	0.10	0.003	<.0001
Sliced Raw Fish and Eel (special fish)	0.03	0.001	0.03	0.001	0.05	0.001	<.0001
Dog meat	0.01	0.001	0.01	0.001	0.02	0.001	<.0001
Fish	0.29	0.004	0.27	0.005	0.31	0.006	<.0001
Anchovy	0.37	0.004	0.25	0.005	0.24	0.006	<.0001
Seaweeds	0.49	0.005	0.53	0.007	0.45	0.008	<.0001
Shellfish Seafood	0.08	0.002	0.08	0.003	0.15	0.003	<.0001
Milk/Yogurt	0.48	0.006	0.42	0.008	0.45	0.009	<.0001
Carbonated drink/Ice cream	0.10	0.002	0.07	0.003	0.12	0.004	<.0001
Soy Milk	0.07	0.002	0.06	0.002	0.07	0.003	0.0320
Coffee	3.50	0.034	3.14	0.044	2.90	0.053	<.0001
Traditional beverages	0.12	0.003	0.11	0.004	0.14	0.004	<.0001
Green tea	0.30	0.007	0.28	0.009	0.28	0.011	0.0863
Native fruit	1.16	0.010	0.95	0.012	1.15	0.015	<.0001
Non-native fruit	0.10	0.002	0.11	0.003	0.15	0.003	<.0001
Nutrients							
Carbohydrates, g	308.9	0.3	306.6	0.3	306.2	0.4	<.0001
Fat, g	18.1	0.1	18.6	0.1	18.9	0.1	<.0001
Vegetable Fat, g	8.7	*0.04*	9.1	0.05	8.5	0.06	<.0001
Animal Fat, g	9.6	0.1	9.7	0.1	10.7	0.1	<.0001
Protein, g	47.8	*0.08*	48.5	0.10	49.3	0.13	<.0001
Vegetable protein, g	33.7	0.1	35.1	0.1	34.3	0.1	<.0001
Animal Protein, g	14.6	0.1	14.0	0.1	15.6	0.1	<.0001
Dietary fiber, g	14.4	0.1	15.3	0.1	15.7	0.1	<.0001
Vitamin A, ug RE	440.3	2.5	436.8	3.2	445.7	3.9	0.2013
Vitamin D, ug	1.9	0.02	1.9	0.02	1.9	0.03	0.1624
Vitamin K, ug	137.4	0.8	148.3	1.1	151.8	1.3	<.0001
Thiamine, mg	0.72	0.002	0.75	0.002	0.76	0.003	<.0001
Riboflavin, mg	0.66	0.002	0.67	0.003	0.69	0.004	<.0001
Niacin, mg	9.7	0.03	9.9	0.04	10.3	0.04	<.0001
Vitamin B6, mg	1.15	0.003	1.16	0.003	1.22	0.004	<.0001
Folic acid, ug	377.4	1.32	394.0	1.70	405.0	2.04	<.0001
Vitamin B12, ug	4.26	0.03	3.82	0.038	3.97	0.045	<.0001
Pantothenic acid, mg	4.04	0.005	4.04	0.007	4.09	0.008	<.0001
Biotin, ug	14.2	0.04	14.4	0.05	14.3	0.06	0.1672
Calcium, mg	344.4	1.6	341.21	2.048	344.77	2.45	0.4079
Vegetable calcium, mg	194.9	0.9	217.3	1.1	212.4	1.3	<.0001
Animal calcium, mg	149.2	1.23	123.2	1.58	131.2	1.89	<.0001
Phosphorus, mg	650.1	1.6	648.7	2.1	667.8	2.5	<.0001
Sodium, mg	2452	14.3	2675	18.3	2604	21.9	<.0001
Chlorine, mg	4404	9.7	4311	12.5	4257	14.9	<.0001
Potassium, mg	1767	6.1	1808	7.8	1889	9.4	<.0001
Magnesium, mg	87.7	0.2	90.6	0.3	91.1	0.3	<.0001
Iron, mg	9.19	0.03	9.41	0.03	9.72	0.04	<.0001

Vegetable iron, mg	7.72	0.02	8.11	0.03	8.18	0.03	<.0001
Animal iron, mg	1.46	0.01	1.29	0.01	1.52	0.02	<.0001
Zinc, mg	7.94	0.01	7.99	0.02	8.28	0.02	<.0001
Copper, mg	1.16	0.01	1.17	0.01	1.18	0.01	0.5908
Fluorine, ug	292.8	1.1	282.7	1.4	280.6	1.6	<.0001
Manganese, mg	6.27	0.01	6.27	0.012	6.2	0.015	0.0001
Iodine, ug	158.7	1.9	144.9	2.4	144.8	2.9	<.0001
Selenium, ug	78.8	0.1	79.2	0.137	78.4	0.2	0.0028
Cholesterol, mg	115.6	0.8	111.7	1.1	119.0	1.3	<.0001
Phytate, mg	562.4	1.3	601.1	1.7	590.5	2.0	<.0001
Cereal Fiber, g	4.2	0.01	4.3	0.02	4.47	0.02	<.0001
Vegetable Fiber, g	6.27	0.04	7.11	0.05	7.28	0.06	<.0001
Fruit Fiber, g	1.57	0.01	1.31	0.02	1.51	0.02	<.0001
Glycemic load,	178.3	0.2	175.5	0.3	175.7	0.3	<.0001
Monounsaturated fatty acid, g	5.23	0.03	5.40	0.04	5.71	0.05	<.0001
Polyunsaturated fatty acid, g	4.1	0.021	4.46	0.027	4.4	0.032	<.0001
n-3 Polyunsaturated fatty, g	0.94	0.009	0.94	0.011	0.91	0.014	0.2398
n-6 Polyunsaturated fatty, g	5.37	0.04	5.51	0.05	5.42	0.06	0.1088
Saturated fatty acid, g	6.07	0.03	6.06	0.04	6.35	0.05	<.0001
Soy protein, g	3.75	0.03	4.43	0.04	4.19	0.05	<.0001
Glycemic index,	57.5	0.03	57.1	0.04	57.1	0.05	<.0001
Isoleucine, Leucine, Valine, mg	4263	17.1	4340	21.9	4477	26.2	<.0001
Retinol, μg	0.06	0.001	0.06	0.001	0.06	0.001	<.0001
Vitamin C, mg	45.2	0.29	45.1	0.3	49.8	0.4	<.0001
Vitamin E, mg	4.03	0.02	4.63	0.03	4.52	0.04	<.0001
Carotenoids, μg	6.39	0.05	6.08	0.07	7.35	0.08	<.0001
Flavonols, mg	16.2	0.13	16.9	0.17	17.7	0.20	<.0001
Flavones, mg	1.69	0.011	1.69	0.01	1.83	0.02	<.0001
Flavones, mg	8.18	0.09	6.97	0.12	9.16	0.14	<.0001
Flavan-3-ols, mg	58.3	1.70	50.9	2.19	51.2	2.62	0.0133
Anthocyanins, mg	8.18	0.10	6.13	0.12	9.25	0.15	<.0001
Isoflavones, mg	17.2	0.15	19.6	0.19	19.5	0.23	<.0001
Proanthocyanidins, mg	54.7	0.55	53.3	0.71	57.2	0.81	0.0016
Total Flavonoids, mg	161.7	1.77	153.4	2.28	163.8	2.72	0.0037

*The values are adjusted for age and expressed as mean ± SE; *P* values for differences were determined by general linear modeling (Tukey's multiple comparisons)

Supplementary Figure S1. Seven clusters of food identified by hierarchical cluster analysis in the three cohorts*

*Clusters were obtained by hierarchical cluster analysis based on Spearman correlation coefficients

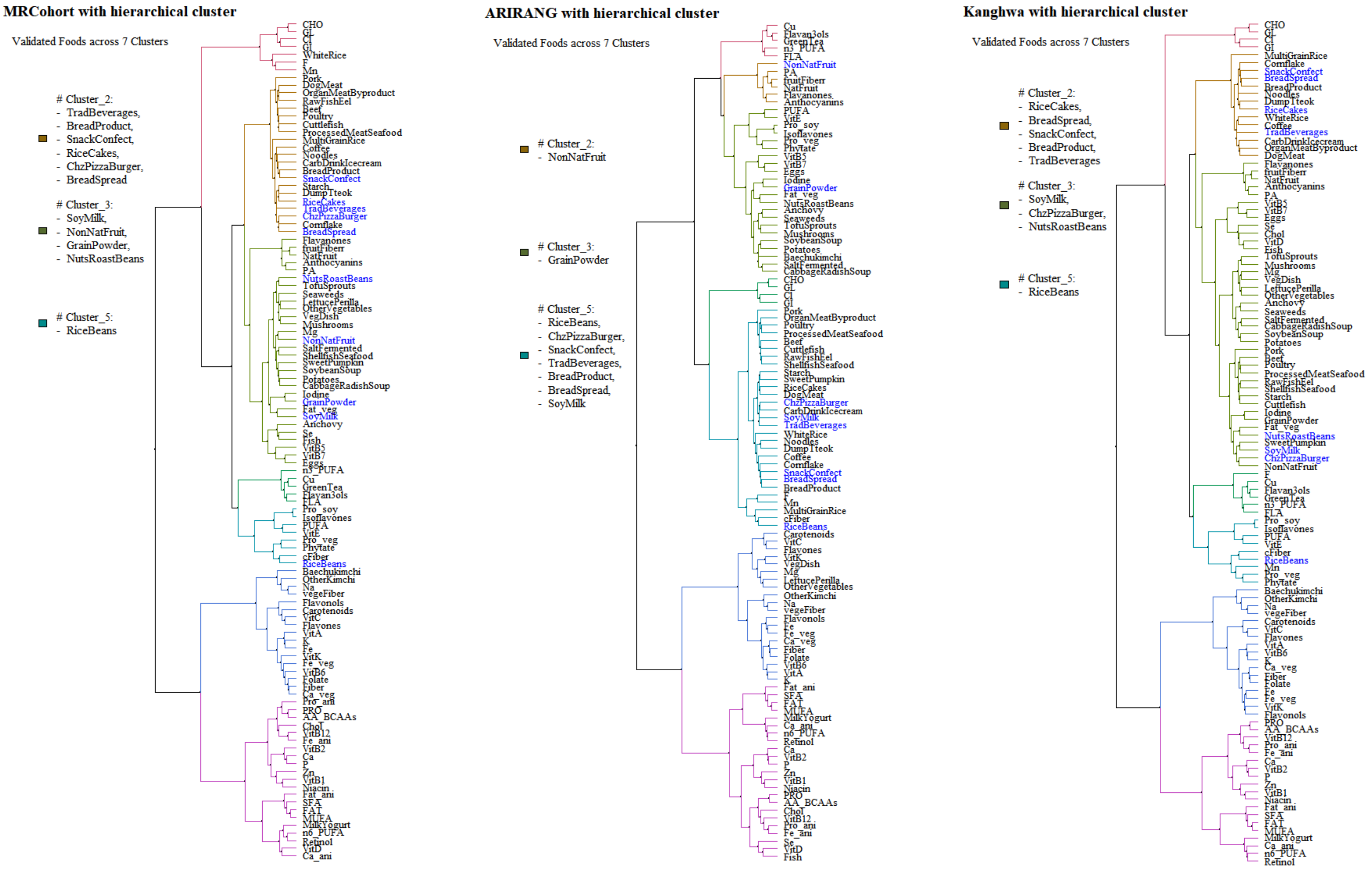

Supplementary Table S3. Clustered 47 Foods and 62 nutrients (Cluster numbers are assigned in the order shown in **Supplementary Figure 1**)*

Group No	Group Name	Cohort Name	Cluster No	Clustered 47 Foods and 62 Nutrients
**Group 1**	*Carbohydrate nutrition factors*	MRCohort	Cluster 1	CHO, Cl, F, Mn, GL, GI, WhiteRice
		ARIRANG	Cluster 4	CHO, Cl, GL, GI
		Kangwha	Cluster 1	CHO, Cl, GL, GI
**Group 2**	*Animal-based nutrients*	MRCohort	Cluster 7	FAT, Fat_ani, PRO, Pro_ani, VitD, VitB1, VitB2, Niacin, VitB12, Ca, Ca_ani, P, Fe_ani, Zn, Chol, MUFA, n6_PUFA, SFA, AA_BCAAs, Retinol, MilkYogurt
		ARIRANG	Cluster 7	FAT, Fat_ani, PRO, Pro_ani, VitD, VitB1, VitB2, Niacin, VitB12, Ca, Ca_ani, P, Fe_ani, Zn, Se, Chol, MUFA, n6_PUFA, SFA, AA_BCAAs, Retinol, Fish, MilkYogurt
		Kangwha	Cluster 7	FAT, Fat_ani, PRO, Pro_ani, VitB1, VitB2, Niacin, VitB12, Ca, Ca_ani, P, Fe_ani, Zn, MUFA, n6_PUFA, SFA, AA_BCAAs, Retinol, MilkYogurt
**Group 3**	***Diverse plant-based foods and Seafood***	MRCohort	Cluster 3	Fat_veg, VitB5, VitB7, Mg, Iodine, Se, fruitFiberr, Flavanones, Anthocyanins, PA, **GrainPowder**, **NutsRoastBeans**, SoybeanSoup, TofuSprouts, Eggs, Potatoes, SaltFermented, CabbageRadishSoup, VegDish, LettucePerilla, Mushrooms, OtherVegetables, SweetPumpkin, Fish, Anchovy, Seaweeds, ShellfishSeafood, **SoyMilk**, DomesticFruit, **NonNatFruit**
		ARIRANG	Cluster 3 (*Group 3a*)†	Fat_veg, Pro_veg, VitB5, VitB7, Iodine, Phytate, PUFA, Pro_soy, VitE, Isoflavones, **GrainPowder**, NutsRoastBeans, SoybeanSoup, TofuSprouts, Eggs, Potatoes, Baechukimchi, SaltFermented, CabbageRadishSoup, Mushrooms, Anchovy, Seaweeds
			Cluster 2 (*Group 3b*)†	FruitFiber, Flavanones, Anthocyanins, PA, DomesticFruit, **NonNatFruit**
		Kangwha	Cluster 3	Fat_veg, VitD, VitB5, VitB7, Mg, Iodine, Se, Chol, fruitFiberr, Flavanones, Anthocyanins, PA, **ChzPizzaBurger**, GrainPowder, **NutsRoastBeans**, SoybeanSoup, TofuSprouts, Eggs, Starch, Potatoes, SaltFermented, CabbageRadishSoup, VegDish, LettucePerilla, Mushrooms, OtherVegetables, SweetPumpkin, Poultry, Cuttlefish, Pork, ProcessedMeatSeafood, Beef, RawFishEel, Fish, Anchovy, Seaweeds, ShellfishSeafood, **SoyMilk**, DomesticFruit, NonNatFruit
**Group 4**	***Rice with beans and related nutrients***	MRCohort	Cluster 5	Pro_veg, Phytate, cFiber, PUFA, Pro_soy, VitE, Isoflavones, **RiceBeans**
		ARIRANG		
		Kangwha	Cluster 5	Pro_veg, Mn, Phytate, cFiber, PUFA, Pro_soy, VitE, Isoflavones, **RiceBeans**
**Group 5**	*Plant-based nutrients and vegetables*	MRCohort	Cluster 6	Fiber, VitA, VitK, VitB6, Folate, Ca_veg, Na, K, Fe, Fe_veg, vegeFiber, VitC, Carotenoids, Flavonols, Flavones, Baechukimchi, OtherKimchi
		ARIRANG	Cluster 6	Fiber, VitA, VitK, VitB6, Folate, Ca_veg, Na, K, Mg, Fe, Fe_veg, vegeFiber, VitC, Carotenoids, Flavonols, Flavones, OtherKimchi, VegDish, LettucePerilla, OtherVegetables
		Kangwha	Cluster 6	Fiber, VitA, VitK, VitB6, Folate, Ca_veg, Na, K, Fe, Fe_veg, vegeFiber, VitC, Carotenoids, Flavonols, Flavones, Baechukimchi, OtherKimchi
**Group 6**	*Green tea, related phytochemicals, and n3-PUFA*	MRCohort	Cluster 4	Cu, n3_PUFA, Flavan3ols, FLA, GreenTea
		ARIRANG	Cluster 1	Cu, n3_PUFA, Flavan3ols, FLA, GreenTea
		Kangwha	Cluster 4	Cu, F, n3_PUFA, Flavan3ols, FLA, GreenTea
**Group 7**	***Fusion diet combining traditional and non-traditional foods***	MRCohort	Cluster 2	MultiGrainRice, Noodles, DumpTteok, **RiceCakes**, Cornflake, **BreadSpread**, **BreadProduct**, **ChzPizzaBurger**, **SnackConfect**, Starch, Poultry, OrganMeatByproduct, Cuttlefish, Pork, ProcessedMeatSeafood, Beef, RawFishEel, DogMeat, CarbDrinkIcecream, Coffee, **TradBeverages**
		ARIRANG	Cluster 5	F, Mn, cFiber, WhiteRice, **RiceBeans**, MultiGrainRice, Noodles, DumpTteok, RiceCakes, Cornflake, **BreadSpread**, **BreadProduct**, **ChzPizzaBurger**, **SnackConfect**, Starch, SweetPumpkin, Poultry, OrganMeatByproduct, Cuttlefish, Pork, ProcessedMeatSeafood, Beef, RawFishEel, DogMeat, ShellfishSeafood, CarbDrinkIcecream, **SoyMilk**, Coffee, **TradBeverages**
		Kangwha	Cluster 2	WhiteRice, MultiGrainRice, Noodles, DumpTteok, **RiceCakes**, Cornflake, **BreadSpread**, **BreadProduct**, **SnackConfect**, OrganMeatByproduct, DogMeat, CarbDrinkIcecream, Coffee, **TradBeverages**

*Bold is validated foods.
† In the ARIRANG cohort, Clusters 3 and 2 were labelled as Group 3a and 3b, based on classifications from other cohorts.

Supplementary Table S4. Stratified IRR (95% CI) of type 2 diabetes according to quartile (Q) of cooked rice with beans and non-native fruit by risk factors.*

Various risk factors for T2D		MRCohort				ARIRANG				Kanghwa			
		T1	T2	T3	*P* interaction	T1	T2	T3	*P* interaction	T1	T2	T3	*P* interaction
**Cooked rice with beans**													
Age, y	< 65y	1.00	0.35 (0.27-0.46)	0.42 (0.32-0.54)	0.4046	1.00	0.22 (0.15-0.32)	0.39 (0.28-0.53)	0.4815	1.00	0.36 (0.24-0.54)	0.34 (0.22-0.51)	0.6351
	≥ 65y	1.00	0.30 (0.22-0.42)	0.30 (0.22-0.42)		1.00	0.26 (0.13-0.50)	0.25 (0.13-0.48)		1.00	0.47 (0.20-1.12)	0.54 (0.25-1.18)	
Regular exercise	No	1.00	0.33 (0.26-0.41)	0.38 (0.31-0.48)	0.7285	1.00	0.22 (0.16-0.32)	0.38 (0.28-0.52)	0.7140	1.00	0.35 (0.23-0.54)	0.35 (0.23-0.53)	0.6343
	Yes	1.00	0.36 (0.24-0.56)	0.34 (0.22-0.53)		1.00	0.26 (0.12-0.58)	0.29 (0.14-0.60)		1.00	0.48 (0.20-1.15	0.44 (0.18-1.06)	
Current smoking status	No	1.00	0.33 (0.26-0.41)	0.41 (0.33-0.51)	0.0761	1.00	0.23 (0.16-0.33)	0.41 (0.29-0.56)	0.1710	1.00	0.38 (0.25-0.57)	0.36 (0.25-0.56)	0.9790
	Yes	1.00	0.36 (0.23-0.56)	0.22 (0.12-0.39)		1.00	0.23 (0.11-0.46)	0.22 (0.12-0.41)		1.00	0.45 (0.19-1.09)	0.33 (0.14-0.79)	
Current drinking status	No	1.00	0.35 (0.27-0.46)	0.41 (0.32-0.53)	0.6038	1.00	0.19 (0.12-0.29)	0.36 (0.25-0.51)	0.2867	1.00	0.41 (0.24-0.68)	0.42 (0.26-0.69)	0.5577
	Yes	1.00	0.31 (0.23-0.43)	0.33 (0.24-0.45)		1.00	0.36 (0.22-0.59)	0.31 (0.19-0.51)		1.00	0.34 (0.20-0.57)	0.29 (0.19-0.51)	
BMI, kg/m^2	< 23	1.00	0.23 (0.13-0.38)	0.42 (0.27-0.64)	0.1236	1.00	0.25 (0.11-0.62)	0.44 (0.21-0.94)	0.7108	1.00	0.48 (0.22-1.04)	0.30 (0.13-0.74)	0.6237
	≥ 23	1.00	0.36 (0.28-0.45)	0.36 (0.29-0.45)		1.00	0.34 (0.25-0.47)	0.22 (0.16-0.32)		1.00	0.36 (0.23-0.55)	0.38 (0.26-0.58)	
DQI-I score	< Median level	1.00	0.36 (0.27-0.47)	0.33 (0.25-0.45)	0.3257	1.00	0.21 (0.13-0.35)	0.43 (0.29-0.65)	0.3276	1.00	0.42 (0.26-0.67)	0.33 (0.20-0.54)	0.4716
	≥ Median level	1.00	0.30 (0.22-0.41)	0.40 (0.30-0.53)		1.00	0.32 (0.21-0.48)	0.25 (0.16-0.38)		1.00	0.38 (0.21-0.67)	0.28 (0.15-0.53)	
Prediabetes	FBG <100 mg/dL	1.00	0.42 (0.29-0.61)	0.46 (0.32-0.66)	0.5822	1.00	0.39 (0.29-0.66)	0.56 (0.34-0.92)	0.1040	1.00	0.63 (0.33-1.20)	0.43 (0.21-0.86)	0.1995
	FBG ≥100 mg/dL	1.00	0.36 (0.28-0.46)	0.37 (0.29-0.47)		1.00	0.19 (0.13-0.29)	0.32 (0.22-0.45)		1.00	0.34 (0.22-0.54)	0.45 (0.29-0.70)	
**Non-native fruit**													
Age, y	< 65y	1.00	0.76 (0.58-0.99)	0.69 (0.52-0.91)	0.7423	1.00	0.77 (0.55-1.07)	0.63 (0.43-0.92)	0.8900	1.00	0.62 (0.40-0.96)	0.76 (0.50-1.16)	0.8780
	≥ 65y	1.00	0.75 (0.55-1.01)	0.61 (0.43-0.88)		1.00	0.64 (0.33-1.23)	0.58 (0.28-1.21)		1.00	0.74 (0.34-1.61)	0.71 (0.26-1.95)	
Regular exercise	No	1.00	0.72)0.58-0.90)	0.66 (0.52-0.84)	0.8166	1.00	0.70 (0.51-0.96)	0.61 (0.42-0.88)	0.6898	1.00	0.76 (0.50-1.14)	0.71 (0.45-1.11)	0.1111
	Yes	1.00	0.82 (0.52-1.30)	0.65 (0.41-1.03)		1.00	0.96 (0.43-2.15)	0.71 (0.29-1.74)		1.00	0.29 (0.11-0.81)	0.77 (0.36-1.66)	
Current smoking status	No	1.00	0.75 (0.60-0.93)	0.69 (0.55-0.87)	0.6057	1.00	0.76 (0.55-1.06)	0.61 (0.42-0.89)	0.7770	1.00	0.56 (0.36-0.86)	0.76 (0.50-1.14)	0.2160
	Yes	1.00	0.75 (0.47-1.19)	0.50 (0.28-0.99)		1.00	0.64 (0.33-1.24)	0.64 (0.30-1.36)		1.00	1.29 (0.55-3.07)	0.76 (0.27-2.18)	
Current drinking status	No	1.00	0.87 (0.67-1.13)	0.69 (0.52-0.91)	0.2385	1.00	0.66 (0.45-0.97)	0.69 (0.46-1.04)	0.2914	1.00	0.58 (0.34-0.99)	0.90 (0.55-1.47)	0.2825
	Yes	1.00	0.60 (0.44-0.82)	0.61 (0.44-0.85)		1.00	0.83 (0.53-1.32)	0.50 (0.28-0.89)		1.00	0.72 (0.42-1.23)	0.60 (0.32-1.12)	
BMI, kg/m^2	< 23	1.00	0.85 (0.53-1.35)	0.91 (0.55-1.49)	0.4534	1.00	0.74 (0.33-1.64)	0.64 (0.27-1.53)	0.9698	1.00	0.69 (0.27-1.80)	0.86 (0.34-2.08)	0.9249
	≥ 23	1.00	0.71 (0.57-0.89)	0.60 (0.47-0.76)		1.00	0.73 (0.53-1.00)	0.61 (0.42-0.87)		1.00	0.63 (0.4-0.96)	0.72 (0.47-1.11)	
DQI-I score	< Median level	1.00	0.72 (0.56-0.94)	0.63 (0.45-0.89)	0.9348	1.00	0.94 (0.63-1.39)	0.74 (0.43-1.25)	0.2293	1.00	0.64 (0.39-1.03)	0.99 (0.58-1.68)	0.5942
	≥ Median level	1.00	0.75 (0.55-1.03)	0.66 (0.49-0.88)		1.00	0.56 (0.36-0.89)	0.53 (0.34-0.81)		1.00	0.74 (0.38-1.42)	0.86 (0.48-1.55)	
Prediabetes	FBG <100 mg/dL	1.00	1.07 (0.75-1.51)	0.69 (0.46-1.07)	0.0174	1.00	0.72 (0.45-1.17)	0.46 (0.26-0.82)	0.2224	1.00	0.50 (0.25-0.99)	0.65 (0.34-1.26)	0.5217
	FBG ≥100 mg/dL	1.00	0.60 (0.47-0.75)	0.61 (0.47-0.79)		1.00	0.82 (0.57-1.18)	0.74 (0.50-1.10)		1.00	0.78 (0.50-1.21)	0.82 (0.52-1.30)	

*Data are expressed as IRR (95% CI). The multivariable model was adjusted for age (years), higher education level (≥ 12 years of education), regular exercise (≥ 3 times/week and ≥ 30 minutes/session), current drinking status (yes or no), alcohol consumption (g/day), and total energy intake (kcal/day).

† *P* values were obtained using modified Poisson regression with a robust error estimator, and interactions with age, regular exercise, smoking status, drinking status, BMI, DQI-I, and prediabetes status were tested using a cross-product term.

Supplementary Figure S2. Correlation Analysis of Validated Foods and Nutrients

*Foods and nutrients depicted in the figure are validated foods. All correlations shown are either between validated foods or between nutrients and validated foods, with a correlation coefficient of 0.2 or higher, within clusters that included at least one validated food.

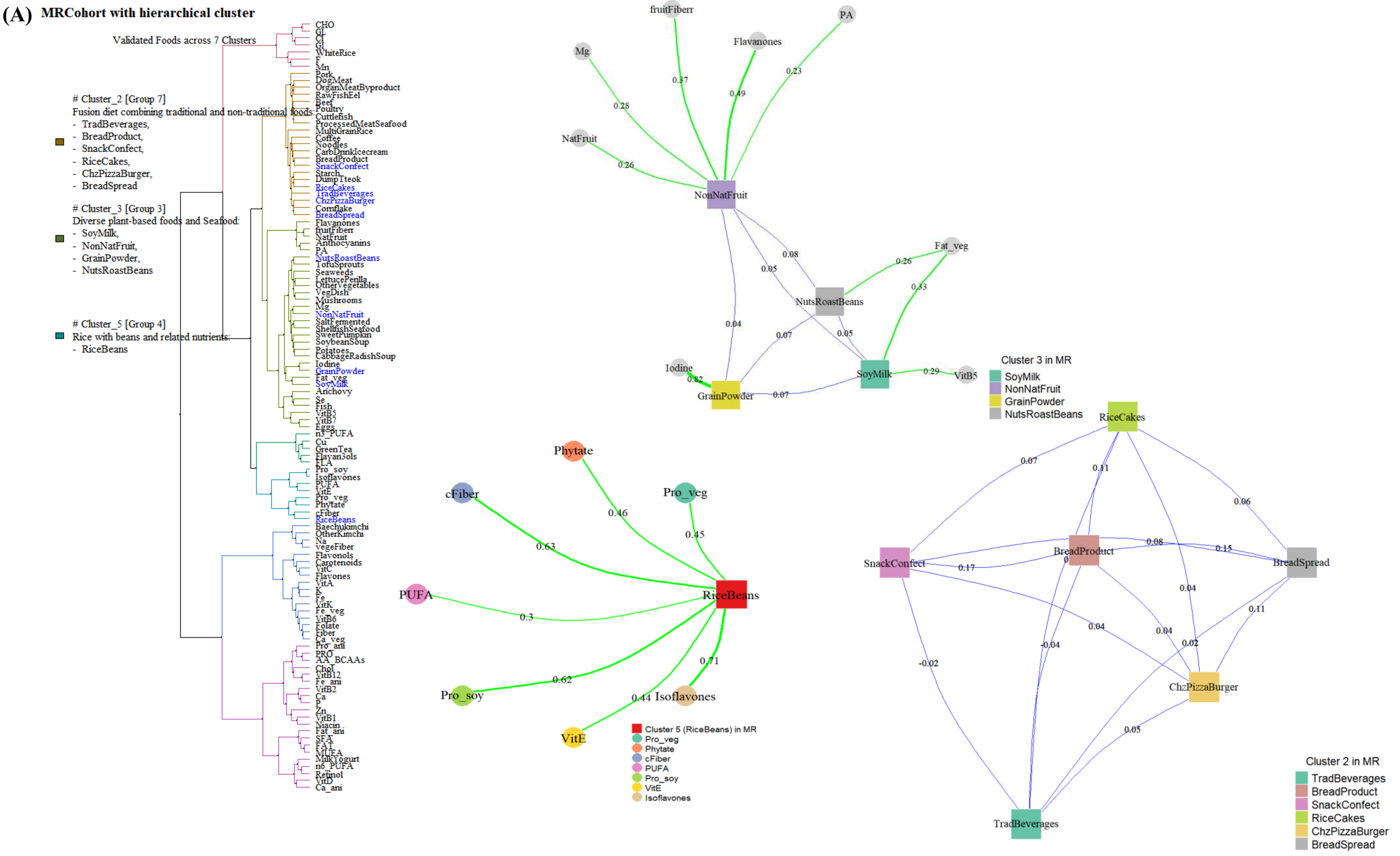

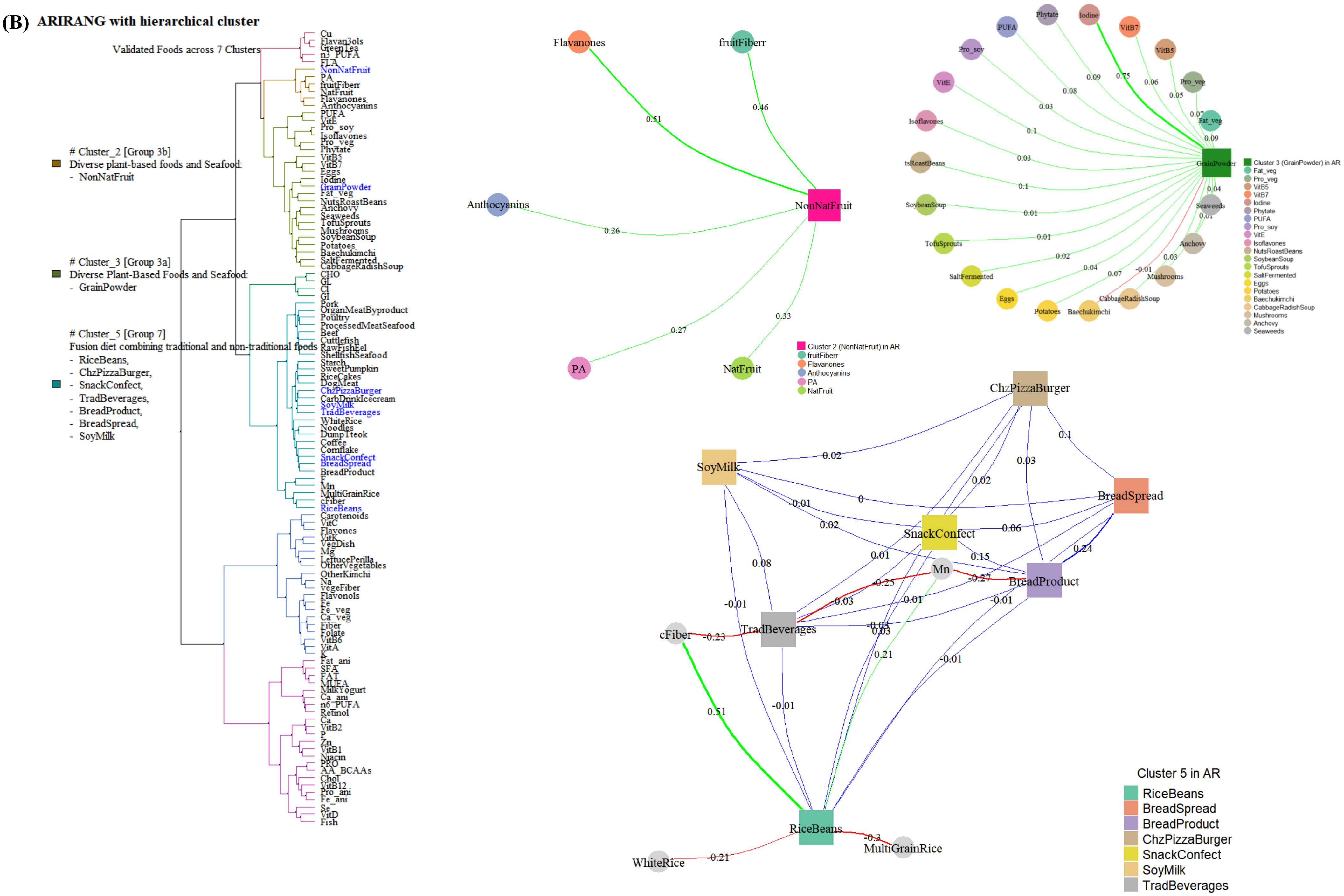
(B) ARIRANG with hierarchical cluster
Validated Foods across 7 Clusters
Cluster_2 [Group 3b]
Diverse plant-based foods and Seafood:
- NonNatFruit
Cluster_3 [Group 3a]
Diverse Plant-Based Foods and Seafood:
- GrainPowder
Cluster_5 [Group 7]
Fusion diet combining traditional and non-traditional foods
- RiceBeans,
- ChzPizzaBurger,
- SnackConfect,
- TradBeverages,
- BreadProduct,
- BreadSpread,
- SoyMilk
Cluster 2 (NonNatFruit) in AR
fruitFiberr
Flavanones
Anthocyanins
PA
NatFruit
NonNatFruit
Cluster 3 (GrainPowder) in AR
GrainPowder
Fat_veg
Pro_veg
VitB5
VitB7
Iodine
Phytate
PUFA
Pro_soy
VitE
Isoflavones
NutsRoastBeans
SoybeanSoup
TofuSprouts
SaltFermented
Eggs
Potatoes
Baechukimchi
CabbageRadishSoup
Mushrooms
Anchovy
Seaweeds
Cluster 5 in AR
RiceBeans
BreadSpread
BreadProduct
ChzPizzaBurger
SnackConfect
SoyMilk
TradBeverages
cFiber
Mn
WhiteRice
MultiGrainRice

(C) Kanghwa with hierarchical cluster

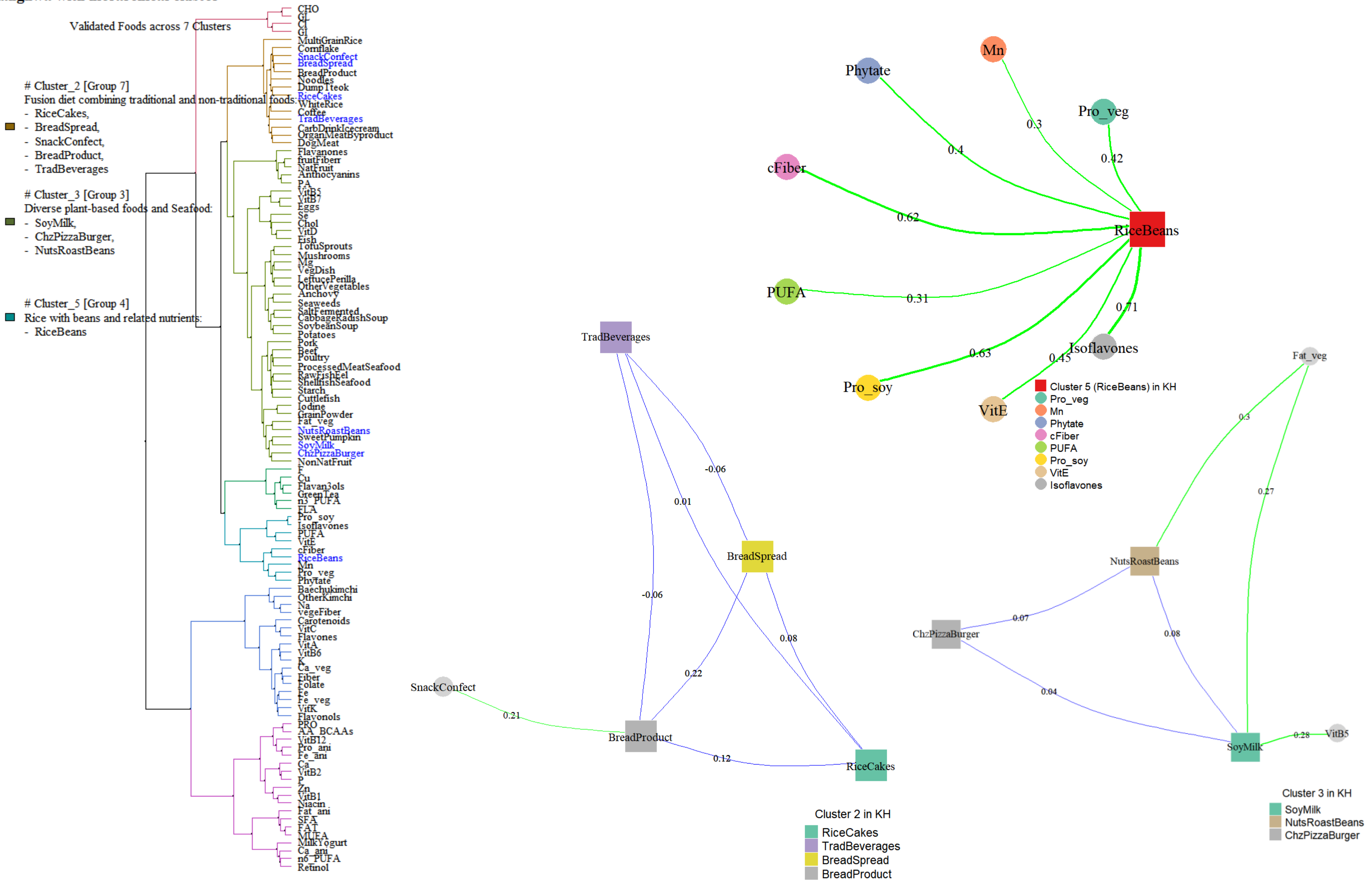

Supplement: Supplementary file 1 [file nutrients-16-03798-s001.zip › nutrients-3270415-supplementary.pdf]
